# Making outcome measures matter: Why should “what matters to people living with dementia” matter to dementia researchers?

**DOI:** 10.1002/alz.70359

**Published:** 2025-06-22

**Authors:** Siobhan T. Reilly, Andrew J. E. Harding

**Affiliations:** ^1^ Centre for Applied Dementia Studies Faculty of Health Studies University of Bradford Bradford UK; ^2^ Centre for Ageing Research Division of Health Research Faculty of Health and Medicine Lancaster University Lancaster UK; ^3^ Division of Health Research Faculty of Health and Medicine Lancaster University Lancaster UK

**Keywords:** Alzheimer's disease, core outcome set, dementia, measurement, outcome

## Abstract

This article provides an overview of evidence to support a call to action for dementia researchers to ensure that “what matters to people living with dementia” should be at the heart of any decision‐making around the choices and design of outcome measures. There have been sufficient reviews observing how the outcome measures that have been used in previous research have not been those that have been valued by people living with dementia or their carers. If researchers continue to use existing measures that are not valued by people living with dementia, they will waste limited research resources by using measures that are not sufficiently sensitive to detect changes that might be attributed to interventions. It is time for researchers to collaborate internationally to ensure that resources are invested in designing and validating new approaches for measurement of psychosocial outcomes for those living with dementia.

**Highlights:**

- Outcome measures that have been used in previous research have not been those that have been valued by people living with dementia or their carers.
- Existing outcome measures have been shown not to be fit for purpose and tend to focus on symptom reduction or broad conceptualizations of quality of life.
- Dementia researchers will need to collaborate internationally to ensure that resources are invested in designing and validating new approaches for measurement of psychosocial outcomes for those living with dementia.

## 1 BACKGROUND

It has been both a privilege and opportunity to be able to highlight the progress on the importance of this topic through presenting a keynote presentation at the Workshop on Clinically Meaningful Outcomes in Alzheimer's disease and related dementias (AD/ADRD) Trials, held on March 12, 2024. This article summarizes that presentation by one of the authors (S.R.) and the discussion afterwards. We hope it will encourage more dementia researchers to critically consider “what matters to people living with dementia” when making decisions on the choices and design of outcome measures. If the outcomes do not reflect what matters to people with dementia, researchers are likely to be measuring the wrong things. The article initially outlines some background detail on the basics of outcome measurement—definitions, literature, and challenges. Then we highlight previous research on the development of a core outcome set for what matters most to people with dementia living at home^1^, ^2^, ^3^, ^4^, ^5^ showing that the outcome measures that have been used in previous research have not been those that have been valued by people living with dementia or their carers. Finally, we outline some discussion, reflections, and implications for research with a focus upon the current state of outcome measurement and possible ways forward and a call to action. There are a variety of definitions of an outcome. It is “a natural or artificially designated point in the care of an individual or population suitable for assessing the effect of an intervention, or lack of intervention.” ^6^ It is also important to recognize the temporal nature of outcomes, which are “a measurable change in health status, sometimes attributable to a risk factor or an intervention.”^7^ A patient‐reported outcome (PRO) is “any report of the status of a patient's health condition that comes directly from the patient without interpretation of the patient's response by a clinician or anyone else.”^8^ And patient‐reported outcome measures (PROMs) “are instruments that are used to measure the PROs, most often self‐report questionnaires.”^9^ The selection of appropriate outcomes, or domains of outcomes, is a crucial part of designing trials and evaluations in ways that minimize bias. Furthermore, outcomes need to be relevant and important to key stakeholders or interested parties making decisions about health and social care, especially in the choice of the primary outcome measure. ^10^ Outcome measures designed to measure change within individuals over time are dependent not only on their reliability and validity, but also on their responsiveness, their ability to detect minimal clinically important differences.^11^

Where might researchers start to look to identify appropriate outcome measures? From our experience of working in this field for over a decade, we identify the following sources, but these are by no means exhaustive:

1. Systematic reviews on the quality of outcome measurement instruments would be a good place to start. For example, one might look in the COSMIN Database for Systematic Reviews to investigate whether a review of interest is available. The COSMIN initiative aims to improve the selection of outcome measurement instruments for research and clinical practice by developing methodology and practical tools for selecting the most suitable outcome measurement instrument. As part of this initiative, COSMIN systematically collects systematic reviews of outcome measurement instruments. Such systematic reviews are important tools for the selection of outcome measurement instruments for research and clinical practice and for identifying gaps in knowledge on the quality measurement properties of outcome measurement instruments.
2. The Core Outcome Measures in Effectiveness Trials (COMET) initiative https://www.comet‐initiative.org/ brings together people interested in the development and application of agreed standardized sets of outcomes, known as “core outcome sets” (COS). These sets represent the minimum that should be measured and reported in all clinical trials of a specific condition, but COS are also suitable for use in routine care, clinical audit, and research other than randomized trials. The existence or use of a core outcome set does not imply that outcomes in a particular study should be restricted to those in the relevant core outcome set. Rather, there is an expectation that the core outcomes will be collected and reported, making it easier for the results of studies to be compared, contrasted, and combined as appropriate, while researchers continue to explore other outcomes as well.
3. The Patient‐Reported Outcomes Measurement Information System PROMIS:  PROMIS (healthmeasures.net) displays a set of reliable and valid patient‐reported outcome measurement instruments for measuring aspects of physical, mental, and social health, which can universally be applied to adults and children with or without (chronic) diseases. However, at the time of writing none are focused specifically on dementia.
4. For clinical practice purposes, the International Consortium for Health Outcomes Measurement (ICHOM) produce standard outcome sets patient‐centered outcomes through value‐based healthcare and have recommended the outcomes “that matter most to persons with Dementia”. This standard set of outcomes includes measures for motor function, communication, sleep, learning disorders, aggression, caregiver burden, and overall quality of life. These are recommended for providers of care around the world to measure and monitor the outcomes, with the ultimate goal of improving the value of care. However, this was developed with minimal input from people living with dementia and used existing conceptualizations of outcomes/domains.

There are a number of reviews and consensus exercises that explore what outcomes have been and should be measured in either care or trials of effectiveness for interventions designed for people living with dementia. Some of these claim to look at what outcomes “matter most,” but there are differences in how this is done. Some reviews are focused on what has been measured previously. For example, a team of researchers aimed to describe the outcome measures used in dementia and mild cognitive impairment intervention studies, with particular interest in those evaluating patient‐centered outcomes of functional performance and quality of life. They reviewed 676 dementia trials and 129 mild cognitive impairment trials identified from the Cochrane Dementia and Cognitive Improvement Review Group's register of studies, ALOIS, representing all registered dementia and mild cognitive impairment intervention studies over a 10‐year period (2004–2014).^12^ What was striking was only 13% of studies measured quality of life. Seventy percent of trials reported cognitive outcomes and 29% measured functional performance. The authors noted that “This focus on cognitive performance questions the extent to which intervention studies for dementia are evaluating outcome measures which are relevant to individual patients and their carers.” And they also noted that “The heterogeneity in measures, use of bespoke tools, and poor descriptions of test strategy all support the need for a more standardized approach to the conduct and reporting of outcomes assessments.”^12^

Similarly, the Real world Outcomes across the Alzheimer's Disease Spectrum for better care: Multi‐modal data Access Platform (ROADMAP) consortium carried out a systematic review of what outcomes are important to patients with mild cognitive impairment or Alzheimer's disease (AD), carers, and professionals.^13^ They identified 32 clinical, practical, and personal outcomes across 7 domains from the 34 included studies. They sought research studies that elicited information from stakeholders, addressing the research question: Which outcomes of AD across the spectrum are prioritized by patients, caregivers, and health care professionals? These included clinical (memory, mental health), practical (ability to undertake activities of daily living, access to health information), and personal (desire for patient autonomy, maintenance of identity) outcomes of the disease. They also identified several additional outcomes that are infrequently assessed in clinical trial settings, including preservation of the patient's personality or the accessibility of health services and disease information. These concepts may be captured by patient‐reported outcome and experience measures that help to evaluate the effectiveness of treatments and assess the quality of care. This may help with improving patient and carer engagement with clinical trials, which in turn may help with recruitment to trials.

Another group of U.S. researchers have conducted an interview‐based study assessing what matters most to patients with or at risk for Alzheimer's and care partners with 60 individuals and care partners across five AD stages (*n* = 12 each); non‐clinically impaired individuals with AD pathology; individuals with mild cognitive impairment and AD pathology; individuals with mild AD; individuals with moderate AD and their care partners, and care partners of individuals with severe AD.^14^, ^15^ This is part of the “What Matters Most (WMM) series,” an initiative to better understand and assess treatment‐related needs, symptoms, impacts, and outcomes of individuals at risk for or living with AD and the care partners of individuals living with AD. The WMM researchers conducted qualitative research with individuals at risk for or living with mild AD and care partners of individuals with more advanced AD to identify a comprehensive set of 42 AD symptoms, impacts, and treatment‐related outcomes that are meaningful to individuals across the AD continuum. The interview guide was driven by the concepts and domains included in the Alzheimer's Disease Assessment Scale–Cognitive (ADAS‐Cog)^16^ and information from the American Psychiatric Association's Diagnostic and Statistical Manual of Mental Disorders. This may explain the emphasis on symptoms and deficits (in memory, communication, concentration, planning, orientation, and so on) and why these findings are not consistent with those from the research described earlier^12^, ^13^ or the research highlighted in the following section, which consults with people living with dementia to formulate the questions asked. The later review^17^ highlighted wider concepts of importance to individuals affected by AD that go beyond the common understanding of “cognition” or “function” alone, reflecting a desire to maintain independence, overall physical and mental health, emotional well‐being, and safety.

Around the same time, our UK research team, as part of Work Programme 3 of the Neighbourhood and Dementia Study, developed a core outcome set for non‐pharmacological community‐based interventions for people living with dementia at home. As a precursor to this, we reviewed previous exercises that have sought to attain consensus on outcomes and measures in dementia care and research.^13^, ^18^, ^19^, ^20^, ^21^, ^22^ There was a great deal of variation in the outcomes recommended. We noted that people living with dementia were rarely consulted. Most prior consensus exercises place more emphasis and weight upon the participation of professionals relative to people living with dementia, so it was unclear if outcomes being recommended are important to people living with dementia. It seemed that the choice of outcomes may be driven more by expectations of the health care professionals and the research community,^23^ and this is continued in a more recent consensus exercise with only one person living with dementia participating.^24^ We also noticed that recommendations about outcomes were heavily shaped by existing conceptualization of what constitutes an outcome, domains, or items within. If the starting point in these exercises is too constrained by existing, and in some cases quite dated, thinking, it follows that some researchers may not consider whether these existing conceptualizations actually reflect what people with lived experience think is important.^24^ There are a number of other challenges when measuring outcomes for people living with dementia in research. These include: the heterogenous profile of the condition; the variety in residential settings of people living with dementia; the high degree of variability of outcome measures and tools. These all prevent comparisons of effectiveness across studies. Another challenge in dementia care is balancing the outcomes for people with dementia and their carers, especially where these may provide conflicting pictures. In addition, there is a broad range of outcome measures with only a small proportion being used more than once.^23^ How do we really get trialists and researchers to decide on the same outcome measures, or what the measures should be?

## 2 THE DEVELOPMENT OF A CORE OUTCOME SET FOR WHAT MATTERS MOST TO PEOPLE WITH DEMENTIA LIVING AT HOME

One approach that has a lot of potential to address these challenges in outcome measurement is the development of a core outcome set. The idea of a core outcomes set is simple. You undertake research to attain consensus from the key stakeholders on what is most important, or core, and then you review the existing measures against these core outcomes. The output is a recommendation of what outcomes should be measured as a minimum (possibly alongside other outcomes or measures), and how to measure them across all trials within the scope of the core outcomes set. This enhances comparability, responsiveness, and relevance of research, and it also reduces waste. It is important to note that studies of core outcomes sets require updating periodically and further testing and refining that reflect the views of people from diverse different social and cultural groups. At the time of writing, there are 19 studies that are classified as dementia‐related core outcome sets published and listed in the COMET initiative database. One of these was Work Programme 3 of the Neighbourhood and Dementia Study; the aim of this study is to establish an agreed standardized core outcome set (COS) for use when evaluating nonpharmacological health and social care interventions for people with dementia living at home. We had an underpinning value of co‐research philosophy, and we embedded the participation of people living with dementia and their care partners as co‐researchers throughout the study to design creative and inclusive methods and processes.^5^ We had two main research questions in relation to non‐pharmacological community‐based interventions for people living with dementia at home. Which outcomes should be measured from the perspective of people living with dementia, their care partners, health and social care professionals and policymakers and service commissioners, and research leaders, and how should these outcomes to be measured?

We used a three‐phase mixed‐methods study design, and conducted interviews or focus groups with people living with dementia and a systematic literature review in Phase 1 (Table 1). We identified possible outcomes, and then reduced these down to a manageable number and reworked the outcomes into a suitable format so respondents could answer questions on them in Phase 1, which consisted of a Delphi survey and consensus meeting. The study protocol describes the inclusion criteria, and recruitment and consent process for all participant groups; we recruited from the UK.^4^ The 54 outcomes that made up the Delphi survey^3^ did not include outcomes relating to care partners, delivery of care or processes, or costs or economic‐related outcomes. Of the 288 respondents who rated the importance of the outcome items in Round 1 (21 people living with dementia, 58 care partners, 137 relevant health and social care professionals, 60 researchers, and 12 policy makers), 246 completed Round 2 (85% response rate). For our consensus meeting, we enlisted the support of the late Tony Husband (the renowned cartoonist and illustrator), as we wanted to make the findings accessible to as many people as possible. He documented the discussions at the consensus meeting (https://www.bbc.co.uk/news/av/uk‐england‐lancashire‐49049562). Twenty participants attended the consensus meeting.

**TABLE 1:** Number of outcome items identified in each phase of Work Programme 3 Neighbourhood and Dementia mixed‐methods study design.

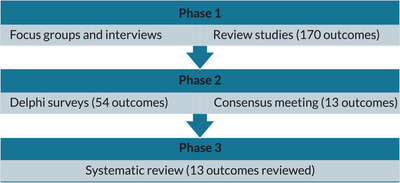

We reached consensus for the inclusion of 13 outcome items to determine what matters most to people living with dementia: positive social relationships; ability to communicate; feeling safe and secure at home; feeling valued and respected by others; being able to have a laugh with other people; being able to engage in activities you enjoy; keeping interested in things you like; being aware of your surroundings; finding your way around a familiar place; being as clean and as comfortable as you would like; not falling; being able to see, hear, and understand; and feeling able to maintain your identity.^1^ We grouped the outcome items into four domains. Self‐managing dementia symptoms, independence, friendly neighborhood and home, and quality of life. In contrast to existing trials where cognitive performance outcomes dominate,^12^ only 3 of the 13 items prioritized in the two rounds of the Delphi survey and the consensus meeting were symptom‐related outcomes. It is important to note that after rating the importance of each outcome in the second round of the Delphi survey, participants were able to change how they rated the outcomes. After being given access to how other participants in other participant groups had rated the outcomes, there were actually a lot of changes recorded. So, 76% came from professional groups and 65% of these changed how they rated the outcomes to align themselves to the views of people living with dementia.

**FIGURE 1:**
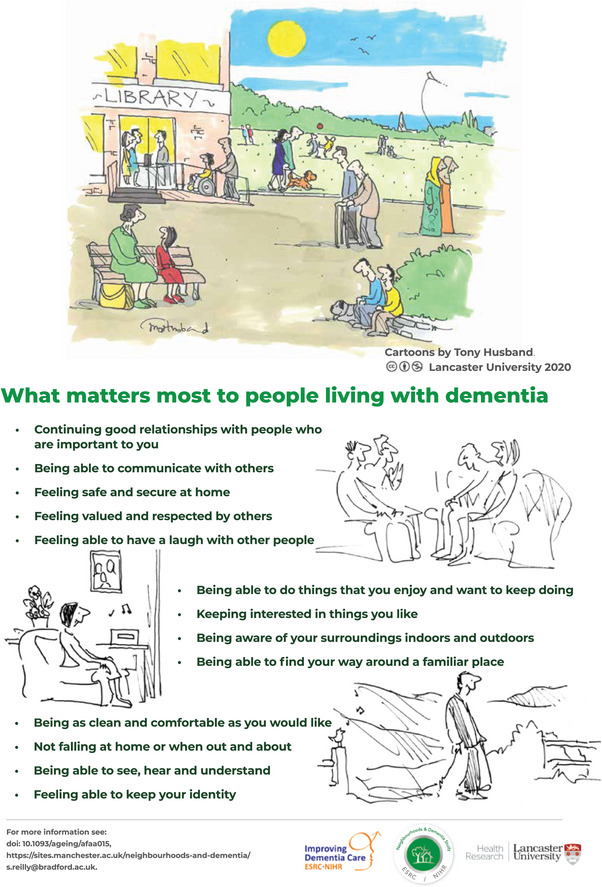
What matters most to people living with dementia.

Given the lack of meaningful participation of people living with dementia in previous consensus exercises, we would suggest that not only did we develop a meaningful method of creative data collection and capture, but also an approach that gave equal weight to the views of people living with dementia and privileged their experience. The final set of core outcome items highlighting what matters most to people living with dementia is presented in Figure 1.

Many of the core outcome items have substantive overlap with the emergent concept of social health, which is a shift in focus from symptoms deficit and disability toward capacity and the potential of the person with dementia.^25^ This is consistent with the work of researchers in the Early detection and timely INTERvention in DEMentia (INTERDEM) network who initiated the Social Health And REserve in the Dementia patient journey (SHARED) project, which is designed to uncover the links between social and brain factors connected to cognitive decline. It is the first international effort to harmonize work on social variables and examine their link to the onset and course of cognitive decline. Social health is a driver for stimulating the use of cognitive reserve through the active facilitation and utilization of the individual's capacities and those of the social environment that slow cognitive impairment or maintain cognitive function in old age. Cognitive reserve is the brain's ability to use brain networks more efficiently or to recruit alternative networks in the presence of pathology. Having a strong social health is associated with reduced risk of cognitive decline and dementia,^26^, ^27^, ^28^, ^29^, ^30^, ^31^ and poor social health is a risk factor.^26^, ^32^ Combinations of social health characteristics have a cumulative effect on cognitive functioning.^33^, ^34^

In phase two of our study, we conducted an extensive systematic literature review to identify outcome measurement instruments that have been used previously in dementia care research to determine how, or even if, the outcome items can be measured. We looked at English‐language questionnaires that people living with dementia complete themselves as subjective questions and are used in social research in the community. We did not include drug trials or those designed for clinical or institutional care settings. We extracted outcomes from 357 sources and identified 347 potential measures. Seventy‐six measures met our inclusion criteria. In order to identify how our 13 outcome measures should be measured, the first step was to ascertain whether the 76 measures were relevant for our purpose; do they reflect any of the 13 core outcomes? To do this, we undertook a screening exercise where we simply compared each of the 76 outcome measurement instrument items with each of the 13 core outcomes, trying to identify where there was a good match. We did this for each item on a four‐point rating scale: very good, adequate, doubtful, and inadequate. If 85% of items in any of the outcome measures had been rated > adequate, we would have then further assessed content validity, internal consistency, and responsiveness. There were a lot of inadequately rated items on most of the instruments and none of the outcome measures reached this standard.

We found an incredible level of dissonance between the core outcome items and the contents of these outcome measurement instruments. Our criterion for relevance was the number/percentage of COS items covered in the instrument. No instruments met the face validity threshold of 85% of items having at least adequate face validity with single or multiple COS items. The majority of instruments had extremely low relevance in respect to face validity with COS items, and some had no relevance. The Engagement and Independence in Dementia Questionnaire (EID‐Q) was found to have the most overlap with the scope and focus of the COS items.^35^ The EID‐Q is a relatively new instrument and was rated as adequate or very good for 7 of the 26 outcome items representing: meaningful activities; losing interest; personal hygiene and cleanliness; a sense of who you are; communication; importance of relationships; and feeling valued and respected by others.

Many of the most frequently used and known measures fared poorly. One example is the measure of health‐related quality of life in dementia (DEMQOL).^36^, ^37^, ^38^ This has been through a rigorous development over 20 years ago. It is the only self‐report quality of life measure that is recommended for use in the UK National Institute for Health and Care Excellence (https://www.nice.org.uk/guidance/qs184) quality standards that should be considered when commissioning or providing dementia services. The DEMQOL has 29 items, of which only 2 of outcome measure instrument items were considered relevant to our 13‐item core outcome set, and only 2 items in our core outcome set were deemed overlapping (adequate or very good) to the DEMQOL items.

## 3 REFLECTIONS AND IMPLICATIONS FOR RESEARCH

Existing outcome measures used in community‐based intervention studies do not reflect what key stakeholders deem to be important, and this was echoed after this keynote presentation at the Workshop on Clinically Meaningful Outcomes in AD/ADRD. David Cella noted during his presentation later in the Workshop that he had checked if the 13 items were represented in the United States National Institutes of Health's Patient‐Reported Outcomes Measurement Information System (PROMIS), which represents a state‐of‐the‐science model for standardized PRO assessment of health‐related quality of life.^39^ PROMIS includes over 300 measures of physical, mental, and social health for use with the general population and with individuals living with chronic conditions; however, it was not developed for people with dementia. He noted that not all of the 13 outcome items could be addressed within PROMIS and maybe this can be a moment in time where the research community commits to developing positive outcome values. This echoed our finding that some of the 13 outcome items were not represented in the outcome measures in the trials we looked at in the first phase of our research.^2^ For example, feeling safe and secure, feeling valued and respected by others, and having a laugh, and continuing good relationships with people who are important to you.

Our research highlights that existing outcome measurement instruments do not really reflect what key stakeholders value. Could these findings help to explain the lack of or minimal positive findings in recent trials? There have been a number of trials of psychosocial interventions for people living with dementia, where there were indications of improved psychological well‐being in those who received the intervention, but either the primary or the secondary outcome measures did not indicate a positive difference. One example is the journeying through the dementia psychosocial intervention study.^40^ The authors note that the lack of positive findings was due to existing outcome measures being used in the trial that were not sufficiently sensitive to detect changes that might be attributed to interventions of this kind. They also note that new approaches are required for measurement of psychosocial outcomes for those with mild dementia. In addition, most of the secondary outcome measures had not been validated in dementia populations due to an absence of available instruments to measure positive outcomes in people with dementia, and they might have been insensitive to differences or changes in outcomes over time or in health status. This might explain some of the small differences observed between the treatment groups. Use of the existing instruments, although unavoidable, was a limitation of the trial. There were similar issues in another study, the Goal‐oriented cognitive rehabilitation for early‐stage Alzheimer's and related dementias: the GREAT RCT. ^41^ This showed that the qualitative findings were at odds with the results from the secondary outcome measures. The intervention may have provided wider benefits that were not detected by standardized measures. Similarly, in the REMCARE trial of reminiscence groups,^42^ there was a lack of positive findings, and this stands in contrast to the reported enjoyment and benefits reported by participants and facilitators. The authors also recommended that interventions for people with dementia should be evaluated in relation to their immediate, within‐session effects, rather than focusing on longer‐term changes.^43^ They refer to an older smaller evaluation of an art gallery access program that went beyond many dementia activities.^43^ They illustrate the problem of outcome measurement that despite no evidence for lasting effects, all participants involved wanted the program to continue, highlighting the sentiment that the activities were deemed highly worthwhile in the moment. The perspective study of Gilmore‐Bykovskyi et al. helpfully outlines four directions to expand and integrate what matters most to individuals living with AD/ADRD into trial outcome evaluation.^44^

The emphasis on the 13 outcome items is also borne out in a couple of recent Dutch qualitative studies. The first study explored how people with dementia in the Netherlands experience their everyday lives, providing insight into what is important to them to live the best they can at home.^45^ A co‐researcher group of seven people with dementia was consulted during the analysis. Six dimensions of what matters most in everyday life were identified: (1) Engaging in meaningful activities, which included routines, household chores, leisure, day activities, and volunteering or work; (2) Keeping a sense of connection, in relationships within the home, with family, friends, groups, and the neighborhood; (3) Having a sense of belonging, which included attachments inside and outside the home, and to cherished objects; (4) Connecting to self, which included the ability to reflect on past experiences, live in the present moment and anticipate the future; (5) Adjusting to ongoing changes, which included alterations in sensory perceptions, perceptions of the physical environment, and navigating shifts in interpersonal dynamics; and (6) Being open to help and support, from professionals, community, and society. The authors note that for people with dementia, everyday life is a continuous balancing act between what matters most and what can be achieved daily. This is not only related to dementia but is also embedded in the wider perspective of life history, relational networks, and the physical environment. This study highlights the importance of identifying what matters most to people with dementia to provide person‐centered support.

Another Dutch study^46^ examined how people living with early dementia realize their well‐being, without treating dementia as a central concern in the conversations or methods. They included people who were able to realize high levels of well‐being. The two main themes focused upon how participants attained well‐being: (1) living a fulfilling life and (2) having a positive attitude toward life by means of engaging in activities, engaging with others, appreciating the good things in life, and coping with difficulties in a positive way. It is difficult to see how the existing outcome measures could capture these concepts. Similarly, others more recently have commented that “the systematic nature of traditional methods and goal‐oriented well‐being definitions does not fit with dementia's changeability, which requires more regular renegotiation in relation to other occurring factors that unfold in a given moment. By rejecting well‐being as a goal or scaled outcome, the value of in‐the‐moment engagements of people with dementia can be understood, and the unpredictable nature of their symptoms accommodated for.” ^47^ They encourage researchers to use inclusive, participatory, place‐based, relational, and in the moment methods.

Our systematic review of 76 existing outcome measures revealed a disconnect between those measures and the core outcomes.^2^ Researchers must work with people living with dementia to develop and test new measures that assess outcomes that matter. Other work that has also shown how people with dementia might live well with the condition indicates that asset‐based factors such as self‐efficacy and humor contribute significantly to overall well‐being.^48^ These findings align with the development of an alternative asset/strengths‐based conceptual framework of well‐being in dementia to help guide instrument development and a more precise matching of intervention aims with outcome measurement. ^49^ Although many studies demonstrate that people with dementia can give reliable accounts of their life using existing dementia‐specific quality of life self‐report instruments, these measures do not capture the full range of outcomes that people with dementia consider important.

Can researchers measure clinically meaningful change if the outcome measures do not reflect what matters most to people living with dementia? Clearly we cannot until more work is done. Focusing on relevant outcomes is fundamental for measuring impact and it goes to the heart of evaluation science. Focusing on relevant outcomes is fundamental for measuring impact. Rossi et al., in their highly influential text, noted “We recommend that evaluators invest the necessary time and resources to develop and test appropriate outcome measures. A poorly conceptualized outcome measure may not properly represent the goals and objectives of the program being evaluated, leading to questions about its validity. An unreliable outcome measure is likely to underestimate the effectiveness of a program and could lead to incorrect inferences about the program's impact. In short, an irrelevant or unreliable measure can completely undermine the worth of an impact assessment by producing misleading estimates. Only if outcome measures are valid and reliable can impact estimates be regarded as credible.”^50^ Some estimate that up to 85%–90% of research funding is wasted due to poor use of resources.^51^, ^52^, ^53^ So if we continue measuring irrelevant or unreliable measures that are not valued by people living with dementia, this will continue to undermine the worth of dementia trials by producing misleading estimates and waste limited research resources. Existing outcome measures do not sufficiently measure what matters most to people living with dementia or the concept of social health. They tend to focus on symptom reduction or broad conceptualizations of quality of life. It is surprising that measuring the most appropriate outcomes has had so little research focus given that clinical trials are only as credible as their outcomes. Academics are also faced with the ever‐present real impetus to obtain research funding, measure, and evaluate, which can perpetuate the difficulties with existing measures because there often is only a short amount of time to select the measures for a research funding proposal. Researchers may understandably look at a review or other studies and they may choose particular outcome measures based on those. But there will be many factors to explain the discrepancy, including the difficulties of obtaining funding for the development of outcome measures.

Will these findings help convince dementia researchers why what matters most to people living with dementia should matter to them? Ideally for the field to progress, this work needs to function as a call to action to dementia care researchers. Ideally researchers will work together and avoid duplication of effort and work as an international network to facilitate better research and make progress on person‐centered measurement and evaluation. Can we develop and test new measures rapidly through an international collaborative of researchers to ensure that ethnic and socio‐economic diverse people living with dementia across the progression of disease and their care partners have their voices heard and are central to the research process so that we have meaningful outcomes consistent with what matters most to diverse people living with dementia and their care partners? Can we use technology to support real‐time, in the moment measurement? One of the vehicles for achieving all of this and addressing the challenges in outcome measurement might be to develop a global organization committed to improving outcomes for people living with dementia. This could be modeled on successful established initiatives in other disease areas, for example, Outcome Measures in Rheumatology (OMERACT), a global, volunteer‐driven, not‐for‐profit initiative, aimed at improving outcome measurement in rheumatology. Being able to measure the core outcome set items that are highlighted would herald a paradigm shift for dementia research in the future and also be responsive to what key stakeholders value.

## CONFLICT OF INTEREST STATEMENT

The authors have no conflicts of interest to disclose. Author disclosures are available in the supporting information.

## Supporting information



Supporting Information
